# Socio Economic Position in TB Prevalence and Access to Services: Results from a Population Prevalence Survey and a Facility-Based Survey in Bangladesh

**DOI:** 10.1371/journal.pone.0044980

**Published:** 2012-09-27

**Authors:** Shahed Hossain, Mohammad Abdul Quaiyum, Khalequ Zaman, Sayera Banu, Mohammad Ashaque Husain, Mohammad Akramul Islam, Erwin Cooreman, Martien Borgdorff, Knut Lönnroth, Abdul Hamid Salim, Frank van Leth

**Affiliations:** 1 International Centre for Diarrhoeal Disease Research, Bangladesh (ICDDR, B), Dhaka, Bangladesh; 2 National TB Control Programme (NTP), DGHS, Dhaka, Bangladesh; 3 Health Programme, BRAC, Dhaka, Bangladesh; 4 World Health Organization, Dhaka, Bangladesh; 5 Department of Global health, Academic Medical Centre, University of Amsterdam, Amsterdam Institute for Global Health and Development, Amsterdam, The Netherlands; 6 Stop TB Department, World Health Organization, Geneva, Switzerland; 7 Damien Foundation, Dhaka, Bangladesh; 8 KNCV Tuberculosis Foundation, The Hague, The Netherlands; McGill University, Canada

## Abstract

**Background:**

In Bangladesh DOTS has been provided free of charge since 1993, yet information on access to TB services by different population group is not well documented. The objective of this study was to assess and compare the socio economic position (SEP) of actively detected cases from the community and the cases being routinely detected under National Tuberculosis Control Programme (NTP) in Bangladesh.

**Methods and Findings:**

SEP was assessed by validated asset item for each of the 21,427 households included in the national tuberculosis prevalence survey 2007–2009. A principal component analysis generated household scores and categorized in quartiles. The distribution of 33 actively identified cases was compared with the 240 NTP cases over the identical SEP quartiles to evaluate access to TB services by different groups of the population. The population prevalence of tuberculosis was 5 times higher in the lowest quartiles of population (95.4, 95% CI: 48.0–189.7) to highest quartile population (19.5, 95% CI: 6.9–55.0). Among the 33 cases detected during survey, 25 (75.8%) were from lower two quartiles, and the rest 8 (24.3%) were from upper two quartiles. Among TB cases detected passively under NTP, more than half of them 137 (57.1%) were from uppermost two quartiles, 98 (41%) from the second quartile, and 5 (2%) in the lowest quartile of the population. This distribution is not affected when adjusted for other factors or interactions among them.

**Conclusions:**

The findings indicate that despite availability free of charge, DOTS is not equally accessed by the poorer sections of the population. However, these figures should be interpreted with caution since there is a need for additional studies that assess in-depth poverty indicators and its determinants in relation to access of the TB services provided in Bangladesh.

## Introduction

The association between tuberculosis (TB) and poverty is well documented. Several studies and reviews concluded that poverty not only exposes a person to more TB infection [1]–[8] but also influences all aspects of the TB disease process [9]–[12]. It has been documented that poverty is associated with delayed care seeking [13], [14], late diagnosis [13], [15] progression of the disease [16], delayed initiation of treatment [13], [16], and inadequate follow up [17]–[19]. It is also noted that poverty leads to poor adherence to treatment, more complications and poor treatment outcomes like default from treatment [2], [8], [20]–[23].

On the other hand, TB disease itself induces many consequences and makes the poor poorer. As a result of poverty-related physical illness, extensive malnutrition and subsequent decreased host resistance the poor are likely to have more extensive and severe forms of TB disease and run higher risks of poor treatment outcomes [20]–[23]. Evidence indicates that the damaging effects of TB are catastrophic to those who were relatively poor or marginalized before being infected with TB. TB subsequently pushes the income insolvent into poverty, the food deprived into a condition of further malnutrition [6], [11], [23], [24]. The long course of the disease and treatment make the poor socially vulnerable and deprived and locks them in the poverty stricken condition. The poor suffers more from TB, and TB puts the poor in more vulnerable state [23], [25]. In fact, Poverty and TB are locked in a vicious cycle, as one triggers the other.

One of the reasons of providing diagnosis and treatment free of cost is that everyone with the disease can access treatment when needed particularly the poorer section of the population. But even after nearly 15 years of Directly Observed Treatment, Short course (DOTS) implementation, the global figure does not support this assumption. The World Health Organization (WHO) has reported the global burden of TB as 8.8 million incident cases in 2010 [26], 82% of them are from 22 high burden countries, categorized mostly as low income countries. With a global case detection rate of 65% for all forms of TB, this means that a marked proportion of these cases did not have any access to quality diagnosis and care. In Bangladesh in 2010 a total of 153,892 new cases were identified, with an estimated case detection rate of just 46% for all cases and 70% for new smear-positive cases. More than 80% of the identified TB cases were from rural areas [26]–[28].

In 2011, Bangladesh was one of the 22 high TB burden countries, having an estimated prevalence of 411 (188–671)/100,000 population [26]. Bangladesh has made considerable progress in DOTS implementation since its adaptation in 1993. The country achieved a 100% DOTS coverage in 2003, the treatment success rate is persistently above 90% from 2000, and case detection rate for new smear positive pulmonary TB above 70% since 2006 [27], [28]. Despite all these successes, evidence on health care utilization, particularly accessing DOTS by socioeconomic groups is scarce. Case notification data segregated by socio economic strata is not available, but inequity in accessing services in other sectors of the health services [29]–[31] suggests that inequity in service utilization might also be a challenging issue for the TB control programme. Such information is needed to improve the implementation and coverage of National Tuberculosis Control Programme (NTP) Bangladesh in more effective and equitable ways. It has been recognized that TB control needs to focus beyond therapeutic strategies to include poverty [2], [8] and tackling the social determinants of TB [2]. National programmes are urged to adopt strategies to address these issues by actively identifying poor and vulnerable populations and facilitate access to diagnosis and treatment to them [8].

In the recently conducted national TB prevalence survey in Bangladesh [32], emphasis therefore had been given to collect information on socio economic position (SEP) of the participants and the TB cases detected in order to assess the relationship between SEP and TB in the country. To assess whether the NTP actually reaches the lower echelons with regards to SEP in the population, we concurrently assessed the SEP from TB cases passively detected by the NTP, and the cases detected actively under a prevalence survey [32].

## Methods

Prevalent TB cases were derived from the national tuberculosis prevalence survey carried out between 2007 and 2009. The objective of this survey was to assess the prevalence of smear-positive TB in Bangladesh. This was a multi stage community based cluster survey including 40 randomly selected clusters, 20 from rural and 20 from urban areas throughout the country. The reason for including equal numbers of clusters from both rural and urban areas was a programmatic decision. It was deemed strategically not wise to put more focus on rural areas than on urban areas, given the large number of stakeholders within TB-control activities in Bangladesh. By including equal number of clusters, we could satisfy all of these stakeholders. From the start we knew that this decision would mean that we had to adjust for unequal sampling probabilities in the analysis, as we did. By including appropriate weights to each included individual, the study population became representative for the target population at large. The methodology is therefore valid, albeit not statistically efficient.

The sample size in the survey was based on the assumption that the prevalence of smear positive pulmonary TB cases varied between 100 and 200 per 100,000 population. It was therefore expected to identify 50 to 100 cases in a sample of 50,000 individuals, or on average 2 cases per cluster. About 52,000 persons ≥15 years of age from 21427 randomly selected households participated in the survey. A detailed description on methods and sample size was published elsewhere [32]. Socioeconomic information was collected from all these households with the household heads being most often the main responders.

We considered an ideal ratio of cases versus controls at 1∶4, which would mean including 8 passively identified cases per cluster. Given the limited time in the cluster this was deemed not feasible, we therefore reduced the ratio to 1∶3 and included 6 controls per cluster, giving rise to 240 cases passively detected under NTP.

Routinely diagnosed TB cases or passively detected control cases were obtained from the most recent registered TB patients in the sub-district TB diagnostic centre of the selected survey clusters. These patients were newly detected smear-positive cases currently under any phases of treatment at a given point of time. The DOTS centers represent the only available place for TB treatment and care under the national programmes in the selected district. The selected patients are therefore a valid representation of passively identified TB cases in the country.

### Outcome Measures: SEP Measurement

Socioeconomic position was estimated by determination of a household asset score based upon ownership of consumer items including home utensils (such as television, bicycle etc.), utilities in home (Bed, Wardrobes), and dwelling characteristics (source of drinking water, sanitation facilities, building materials), that are related to wealth status [33]. Assets were assessed by questionnaire or by direct inspection. The assets score methodology, a composite wealth index, used in this study was developed and tested by the World Bank through the Demographic Surveillance system (DHS), and is used in many countries, including Bangladesh, to estimate inequities in household economic condition, service utilization and health outcomes [34].

To calculate the assets score, each asset item was noted as present or absent from the household. This information was included in a principal component analysis to derive at the weights to be applied to each variable within the calculation of the overall assets score. We used the first principal component only since this account for the maximum possible variability in the data. With each asset assigned a weight, we calculated for each household the assets score based on the presence of assets, in such a way that the overall distribution of assets scores had a mean equals to zero.

The final assets list was reduced from initial 31 to 17 after a careful selection process. We deleted the assets that were present in very small (<20%) or very large number of households (>80%) because they would not provide much discriminatory power to the analysis. We also deleted those assets that had more than 10% missing values because inclusion could lead to skewing of the data. We preferred this over deletion of assets based on a low weight of the asset in the analysis because is less data drive.

We then deleted the few assets that can be seen as a proxy for urban area: that turned out to be “piped water in the house”, “piped water outside house” and “has motorcycle”. The urban effect was additionally addressed by including the urban/rural variable into the multivariable model assessing case detection by SEP.

We did not exclude assets with a potential direct health effect, because we were not assessing the risk of TB in multivariable analysis. Therefore there was not fear of including such an effect twice (as an asset and as a direct risk factor).

A single asset score was developed for all sample households from the rural and urban population. Within the setting of a prevalence survey, the assets score methodology is the recommended approach to incorporate measures of SEP without jeopardizing overall survey objectives [35]. The information of SEP within the survey population was collected during the house-to-house census at the start of the survey. Routinely identified TB patients from the diagnostic centers were visited at their home to obtain this information. The total distribution of assets scores was divided into quartiles from one (lowest) to four (highest). The grouping of households into broad socio economic categories is mostly conventional, based on assumption that SEP is uniformly distributed but occasionally could be data driven [36]. Using quartiles provided the best representation of the relationship between SEP in the study, as tertiles showed marked clumping of cases in the middle category, and quintiles provided difficulties for the small number of actively detected cases in the survey. Also, interpretation of data in quartiles is straightforward with the four categories splitting equally into two groups of upper and lower SEP.

Sputum positive tuberculosis was measured by direct sputum smear microscopy of at least two sputum samples from all participants regardless of symptoms, using fluorescence microscopy at field level laboratories. In line with the national algorithm, TB was diagnosed when two sputum samples were smear positive, or when one sample was smear positive with in addition a chest X-ray suspected of TB.

### Analytical Approach

Data was entered and analyzed using Stata statistical software (Release 10.0, Stata Corporation, College station, TX USA). TB prevalence estimates were based on an adjusted weighted analysis taking into account the survey design and attrition in the survey, using complex survey analyses techniques. Prevalence of TB was stratified by quartiles of SEP in the total survey population. The SEP of TB cases identified in the survey and those identified by the NTP was projected on the quartiles of SEP form the general population for comparison.

The SEP distribution of the survey cases and the NTP cases was compared using a logistic regression approach in which the outcome variable was defined as “low SEP”. A case was considered to have a low SEP when the household SEP was classified as being in the lowest 2 quartiles. Explanatory variable were age (dichotomized at 45 years), sex, urban/rural setting, and type of case (survey or NTP). A p-value <0.05 was considered statistically significant for main effects, while p<0.1 was used for interaction terms.

### Ethical Approval

This study was approved by the ministry of health and family welfare of Bangladesh, the Research Review Committee (RRC) and Ethical Review Committee (ERC) of the icddr,b, a multi disciplinary international research institution situated in Dhaka, Bangladesh. Written informed consent was received from all participants.

## Results

A total 21,427 households were included in the survey, equally from 10688 (49.8%) rural and 10739 (50.2%) urban areas. In total 52,098 adults ≥15 years participated in the survey of which 27,895 (54%) were female. About three quarter (73%) of the survey participants was between 15–44 years of age, 23.6% had no education, and 41% were house wives. The major categories of occupation included agriculture related works (8.8%), employed in the non-agriculture sector works (fishermen, Manual laborer etc.) (14.7%), sales and services (vendors, shops, small business etc.) (18.2%) and Dependent and others (including housewives) (58.3%) (Table 1).

**Table 1:** General characteristics of the participants and the cases detected in the survey and under NTP at the time of survey 2007–2009.

	Survey population	TB cases under survey	TB cases under NTP
Characteristics	n (%)	n (%)	n (%)
**Age in years**			
**15–24**	15275 (29.3)	5 (15.2)	62 (25.8)
**25–34**	12446 (23.9)	3 (9.1)	50 (20.8)
**35–44**	10195 (19.6)	6 (18.2)	43 (17.9)
**45–54**	6803 (13.1)	6 (18.2)	32 (13.3)
**55–64**	4081 (7.8)	7 (21.2)	30 (12.5)
**65+**	3298 (6.3)	6 (18.2)	23 (9.6)
**Sex**			
**Male**	24203 (46.5)	24 (72.7)	152 (63.3)
**Female**	27895 (53.5)	9 (27.3)	88 (36.7)
**Residence**			
**Rural**	26052 (50.0)	20 (60.6)	120 (50.0)
**Urban**	26046 (50.0)	13 (39.4)	120 (50.0)
**Education in schooling years**			
**0**	12300 (23.6)	15 (45.5)	55 (22.9)
**1–5**	11657 (22.4)	8 (24.2)	65 (27.1)
**6–10**	17169 (33.0)	6 (18.2)	62 (25.8)
**11+**	10972 (21.1)	4 (12.1)	58 (24.2)
**Occupation**			
**Agri worker**	4605 (8.8)	4 (12.1)	33 (13.8)
**Non Agri Worker**	7652 (14.7)	14 (42.4)	75 (31.3)
**Sales and Service**	9456 (18.2)	6 (18.2)	109 (45.4)
**Dependents and others (Including housewives)**	30385 (58.3)	9 (27.3)	23 (9.6)

Thirty three new smear positive TB cases were detected in the survey population. Of these, 24 (73%) were male, and 20 (61%) were from rural areas. TB cases from the survey were more from middle to senior age groups of 35 to 65 (19 cases). Nearly half of these TB cases (45%) reported not to have any formal education and most them were engaged in non-agriculture sector works (42%). TB cases detected routinely under NTP were more likely to be male 152 (63%), as was also seen in TB cases identified in the survey. In contrast to the TB cases detected in the survey, routine cases were in general from the lower age group of 15–4 years (155; 64.6%), distributed among education categories from no education (23%) to secondary or more education (50%) and most of them were engaged in non-agriculture related works (31.3%) or in sales and services (45.4%) (Table 1).

The adjusted overall prevalence of smear positive TB was 79.4 (95% CI: 47.1–133.8) per 100,000 population of ≥15 years and above. The prevalence of TB showed a clear gradient by SEP quartiles. The prevalence of smear positive TB was 5 to 6 times higher in the lower two quartiles of SEP with a prevalence 95.4 (95% CI: 48.0–189.7) and 118.4 (95% CI: 50.9–275.3) compared to highest quartiles of SEP with a prevalence of 19.5 (95% CI: 6.9–55.0) (Table 2). This gradient in prevalence was also present for the levels of education. Persons having no education had a four times higher prevalence of TB (138.6; 95% CI: 78.4–245.0) compared to persons having the highest education (39.3; 95% CI: 9.4–164.9) (Table 2).

**Table 2:** Prevalence of tuberculosis.

		Prevalence/100,000 (95% CI)		
Characteristics	Number of TBcases detected	Rural	Urban	All
**Overall prevalence**	33	86.0 (47.9–154.3)	51.1 (27.2–94.1)	79.4 (47.1–133.8)
**Sex**				
** Male**	24	134.5 (70.8–255.4)	70.8 (32.9–152.3)	121.7 (69.6–212.8)
** Female**	9	42.4 (11.4–157.7)	31.0 (12.2–78.7)	40.3 (13.4–121.4)
**Age in years**				
** 15–24**	5	47.8(15.5–146.8)	24.1 (5.6–103.1)	43.0 (16.2–115.0)
** 25–34**	3	58.3(12.0–282.5)	0	46.4 (10.0–215.0)
** 35–44**	6	92.1(36.3–233.5)	41.4 (10.1–170.4)	82.0 (36.0–187.2)
** 45–54**	6	103.0(33.3–317.8)	81.3 (25.1–263.0)	99.0 (39.0–254.3)
** 55–64**	7	212.8(92.7–488.1)	135.6 (31.6–579.6)	201.0 (96.3–418.3)
** 65+**	6	124.5(30.6–504.8)	305.4 (83.4–1112.0)	150.0 (53.5–418.3)
**Asset quartiles**				
**1^st^ (Lowest)**	12	90.6 (41.2–198.9)	169.8 (47.7–602.8)	95.4 (48.0–189.7)
**2^nd^**	13	122.0 (48.3–307.3)	81.8 (27.4–243.8)	118.4 (50.9–275.3)
**3^rd^**	5	37.1 (8.9–155.0)	35.0 (9.9–123.6)	36.6 (11.9–112.5)
**4^th^ (Highest)**	3	0	24.5 (8.5–70.6)	19.5 (6.9–55.0)
**Education in Years**				
**0**	15	143.3 (76.7–267.5)	99.2 (27.9–352.6)	138.6 (78.4–245.0)
**1–5**	8	67.4 (25.2–180.3)	78.5 (29.2–211.2)	69.2(31.2–153.6)
**6–10**	6	61.1 (24.1–154.5)	21.1 (5.0–89.2)	51.8 (22.6–118.6)
**10+**	4	38.0 (4.5–318.1)	42.3 (13.0–137.8)	39.3 (9.4–164.9)
**Occupation**				
**Agri worker**	4	112.2 (39.1–321.7)	0	107.3 (38.4–299.6)
**Non-Agri Worker**	14	191.3(77.3–472.4)	166.3 (79.6–346.8)	187.0 (88.3–395.7)
**Sales and Service**	6	104.6 (22.9–477.2)	60.8 (23.0–160.6)	86.3 (29.7–250.6)
**Dependents and others (Including housewives)**	9	46.7 (15.7–139.0)	19.4 (6.1–61.2)	41.8 (15.7–110.7)

The prevalence was higher in rural compared to urban settings (86.0 vs. 51.1), and three times higher in males compared to females (121.7 vs. 40.3) per 100,000 adult population. The prevalence increased with age being lowest in persons 15–24 years as 43.0 (95% CI: 16.2–115.0) and highest among 55–64 years as 201.0 (95% CI: 96.3–418.3) age groups. These differences persist in both the rural and urban stratum. Occupation wise the prevalence was higher in the working class related either with agriculture 107.3(95% CI: 38.4–299.6) or with non-agriculture works 187.0 (95% CI: 88.3–395.7) compared to the small business or service men 86.3 (95% CI: 29.7–250.6) or of the dependents 41.8 (95% CI: 15.7–110.7), which included a large portion of housewives (Table 2).

The distribution of assets score for the population under survey was 0.38 (95% CI: 0.36–0.38), which was −0.30 (95% CI: −0.30–−0.29) for rural and 1.1 (95% CI: 1.03–1.06) for urban areas, indicating the overall lower score by the general population. From the selected 240 TB cases that were routinely identified by the NTP, 137 (57.1%) were from the two uppermost SEP quartiles, with only 5 (2.1%) TB case detected from the lowest SEP quartile. This distribution was markedly different from the distribution of the 33 TB cases identified in the survey, where 25 (75.8%) were from the lower two quartiles, and only 8 (24.2%) from the two upper most quartiles (Table 3).

**Table 3:** SEP distribution of survey population (Households), TB cases from the survey and cases under NTP.

	Survey population	Cases detected in survey	NTP Cases
Quartiles	n (%)	n (%)	n (%)
**1 (Lowest)**	5348 (24.9)	12 (36.4)	5 (2.1)
**2**	5364 (25.0)	13 (39.4)	98 (40.8)
**3**	5348 (24.9)	5 (15.2)	108 (45.0)
**4 (Highest)**	5367 (25.1)	3 (9.1)	29 (12.1)

Multivariable logistic regression showed that NTP cases were less likely to be classified as having a low SEP compared to survey cases (Odds Ratio [OR]0.27; 95% CI: 0.11–0.69; p = 0.006) (Table 4). This was also true for urban residence (OR: 0.15; 95% CI: 0.08–0.27; p = 0.001). TB cases older than age 45 years were more likely to be classified as having a low SEP (OR: 2.3; 95%CI: 1.31–4.30 p = 0.004). Sex was not associated with low SEP. There were no interactions (effect modification) between type of TB case and sex, age, or residence.

**Table 4:** Unadjusted and adjusted OR with CI of the factors associated with detection of TB cases in poor quartiles of population.

	Quartile					
	Upper	Lower	Unadjusted		Adjusted	
Variables	n	n	OR (CI)	P value	OR (CI)	P Value
**Sex**						
**Male**	90	86				
**Female**	55	42	0.79 (0.48–1.31)	0.378	1.08 (0.60–1.90)	0.773
**Age**						
**15–45**	108	71				
**45+**	37	57	2.30 (1.40–3.90)	0.000	2.30 (1.31–4.30)	0.004
**Residence**						
**Rural**	46	94				
**Urban**	99	34	0.17(0.09–0.28)	0.001	0.15(0.08–0.27)	0.001
**Types of TB case**						
**Actively detected (Survey Cases)**	8	25				
**Passively detected (NTP cases)**	137	103	0.24 (0.10–0.55)	0.001	0.27 (0.11–0.69)	0.006

Multiple logistic model includes a. Age (model with “15–45” age group as reference category b. sex (Male as reference) c. residence (rural as reference and d. Types of TB case (as per mode of detection, actively detected as reference. Quartile has been categorized as lower (1, 2) and upper (3 and 4).

Among cases detected under survey 25 of them belonged to areas where DOTS service is provided by NGO organization like BRAC and Damien Foundation. However only 3 cases were under DOTS, 17 had sought care from other non DOTS sources (9 rural 8 urban) including non licensed providers, pharmacies, other hospitals and graduate private practitioners and half of the cases (50.0%) had no cough at the time of detection (Not shown).

## Discussion

Health systems in most instances are inequitable and follow an ‘inverse care law’ providing more to the rich who need them less than to the poor who cannot afford them. Services provided through the government systems are usually claimed to be universal, but practically the greater share of them are received by the upper quintiles of population [37], [38]. This study focused on the inequalities in accessing free tuberculosis control services (DOTS) in Bangladesh. The findings revealed that nearly 60% of the TB cases detected routinely under DOTS programme belongs to and the upper fraction of the population, on the other hand, 75% of the prevalent cases detected in the survey belongs to lower section of the population.

Notified TB cases in Bangladesh are relatively young and from urban population. These types of patients are less likely to have been classified as having a low SEP. This implies that a large proportion of untreated TB cases remained undetected under routine condition particularly among the poor, particularly in the rural areas. The higher prevalence of TB among the lower quartiles of population also indicates that the poor suffer more and probably delay in detection and treatment.

The availability of Global Fund to fight AIDS, Tuberculosis and Malaria (GFATM) since 2003 enabled augmented advocacy communication and social mobilization (ACSM) and other interventions under NTP to facilitate the currently practiced passive mode of case detection. These activities most likely contributed to the increase in the case notification rate of new smear positive TB cases from 40/100,000 population in 2003 to 74/100,000 population in 2009 [26]. However, potential sociological and economical divide of notification was never reported or addressed. Our data indicates that still poor, less educated and worker class people continues to bear the higher prevalence of the disease. The utilization of DOTS services is linked with adequate knowledge of TB and awareness of DOTS programme, which have been reported to be poor in this part of the world [12], [13], [39], [40]. Targeting the poorer section of population who has less education and less access to media might boost the ongoing ACSM activities.

Achieving wider population coverage of disease-control programmes has been a great health system challenge for the last few decades [38] globally as well as in Bangladesh. This study provides evidence that in case of TB, coverage of DOT does not guarantee that the services are equitably utilized by all sections of the population. Except for some primary care services like immunization, the poor-rich difference seems to be large in other government provided health services in the country [38], [41]. Even nowadays popular universal health coverage initiatives showed that the initial rapid coverage of up to 75%, reach the rich first, the poor have to wait till the threshold is reached. What the poor get is mostly the result of spilling of or percolation of services [38]. In case of TB services coverage in Bangladesh, after nearly twenty years of successful DOTS programme implementation, the lack of TB case detection within the subpopulation with lower SEP raises the question of the proper utilization and acceptability of DOTS in this country. Like in other fields of care seeking, evidences from this study again confirmed that mere availability of free diagnostic and treatment services did not guarantee their utilization [12], [31], [38].

The issue is probably more complex than coverage and utilization. It is often argued that the relationship between TB and poverty is complex and bidirectional. Mere medical intervention alone will not address this relationship adequately. It is necessary to understand the disease from an epidemiological point of view in relation to its social determinants and consequences. The long duration of TB treatment with its associated conditions of absenteeism from the job, or lack of support in the interim period toll heavily on the poor. Poor cannot cope with the situation easily and ultimately default from treatment, take resort to any other affordable and available short cut like buying drugs over the counter for short period, or use other services. The health system itself very often suffer from rapid turnover of experts, running short of supplies and drugs, or sometimes simply lack of initiative in the absence of committed monitoring and support.

Despite country wide coverage of DOTS services by NGOs like BRAC and Damien Foundation, only 3 cases among the detected cases in the survey were under DOTS at the time of identification. This observation raises the question of system bypass, the role and involvement of the private health sector, delay in detection, and probably other programmatic and behavioral factors in the care seeking pathway [12], [13]. There is anecdotal evidence that people in Bangladesh perceive DOTS centres as only TB treatment centres, resulting in TB suspects (subject with prolonged cough) to bypass DOTS centre and attend either the private sector or non government clinics for their initial consultation and diagnosis [12], [13]. In urban areas of Bangladesh, more than 80% of TB suspects first attended a private sector provider, mostly a non-licensed provider for their initial consultation. Only 16% attended any DOTS centre for their symptoms [12]. The huge TB campaign focusing on “seek TB diagnosis when having cough more than three weeks” should take these issues into consideration.

Indirect evidences of private sector preferences, huge out of pocket health expenses at household level (>60% of total health expenditure), and large system delay in TB diagnosis and management, indicate the inefficient utilization of DOTS in the country [42]. Many TB knowledge attitude and practice (KAP) studies conducted in Bangladesh, India and Pakistan largely support the fact that there are still many barriers for the poor peoples to utilize DOTS. In China, India and other parts of the world studies found that even well implemented DOTS could not reach all sections of population [11], [43]–[45]. In fact the financial burden largely weighs heavily on the poorer part of the population and DOTS simply shifts the barriers of expenditures after to before diagnosis [43].

It seems that the classical five components of DOTS [46] may not be sufficient to improve the situation. The Stop TB Strategy does acknowledge the need for attention to vulnerable groups, health system strengthening, engagement with the private sector, and community engagement [47]. However, in addition there is an explicit need for addressing the social determinants of health in relation to TB, to actively reduce barriers for access-to-care, and to provide social support for those in need. These are formidable challenges to be considered but immediate efforts should be taken and directed towards achieving universal and equitable coverage of service throughout the country. How the modulating of social determinants will impact tuberculosis situation is not well documented, but there is indirect evidence that social improvement has a positive effect on health and other development areas. For example microfinance and some other interventions emerged as major strategies to address social conditions like poverty alleviation, women empowerment and overall development in Bangladesh [48]. Properly instituted and targeted Microfinance could also play a major role in addressing the social determinants of TB as the microfinance philosophy contribute to “double bottom line” of financial and social objectives. Our findings support this fact that TB is associated with both. In a recent systematic review, Boccia et al. concluded that cash transfer and microfinance interventions can positively impact TB risk factors, even though only 1 out of 23 studies targeted TB indirectly, while others were related with social well being and improved health care access [49].

The data of this study came from the national prevalence survey which was carried out throughout the country during 2007–09. Therefore, the major strength of the study is that it represents the country population at large by using a valid, albeit not efficient, multi stage cluster methodology. Assets items were directly observed by the trained research assistants and noted in the pretested formats in the field level and a highly motivated supervisory structure was marinated during the whole survey period. Standard operating procedures were followed all the time of data collection. We tried to assess the major pitfalls in using assets as a proxy of wealth and the PCA methodology as an analytical approach.

PCA gives the most reliable results when underlying variables varies across and are well correlated. Extremely distributed variables get either more weight or very low weight producing very high or low standard deviations affecting the results adversely and do not contribute much in differentiating SEP between the groups or households [50]. As a result, it is advised to exclude those items which are very common or very rare in the population. We followed this approach by reducing the included assets as much as possible based on the frequency that assets were reported in the population. Alternately, some authors tried including those variables only significant at 1% level based on factor loadings [51]. We decided not to follow this approach, as it is fully data driven.

Combining geographical variation (Rural and urban) in a single asset index may affect the weights estimated for the variables, as some items may be valued differently between urban and rural location. Including assets that are mainly common in urban areas can overestimate the wealth in urban areas and at the same time mask detailed differences in rural areas. We therefore excluded some of the variables like “piped water in the house”, “piped water outside house” and “has motorcycle” from the analysis. In addition, we have adjusted for urban location in the primary analysis assessing SEP and case-detection. In this analysis the rural population has lower score and asset density which shows that the rural population is relatively poorer than the urban population. In the assessment of case detection and SEP, we therefore have included a variable denoting urban/rural population.

Assets with direct health effect were not excluded from our analysis. This is recommended when the analysis has the focus of assessing risk of a health outcome (i.e. TB) by SEP stratum. If not included, the effect of such a variable is “counted twice”, one in the SEP and one in the multivariable analysis. As our focus was to assess case detection method rather than risk of TB, we did not have to resort to further exclusion of variables.

A potential imitation of the study is related to data collection. In some clusters there were difficulties in assessing the assets on the individual households due to dual or composite ownership of certain household assets (e.g. a motorcycle, TV set etc.), multiple household heads with different assets in a same household in some parts of urban areas and hesitancy or hiding some asset items due to different reasons. As such here is room for ascertainment bias. Given the large number of households and the fact that the misclassification is most likely non-differential, we are convinced that the results are a valid representation of the SEP in the general population.

In conclusion it can be stated that the country-wide covered free DOTS programme mostly serves the richer or middle class population and fails to reach the marginalized population where the prevalence of TB is most prominent. Universal and sustainable coverage can never be achieved without reaching the poor [52]. Many of the TB cases would not have been detected or markedly later if active household search through the survey was not undertaken. Strategies should therefore target the poor and should consider adequate modes of case detection other than the currently practiced passive approach to reach these groups of people from the planning period to the implementation phases of the programme.

## Supporting Information

Figure S1 **Distribution of asset score in the survey population.**
(TIF)
