# Supplementary figures and images for: Socio Economic Position in TB Prevalence and Access to Services: Results from a Population Prevalence Survey and a Facility-Based Survey in Bangladesh
Source: PLoS One. 2012 Sep 27;7(9):e44980. doi: 10.1371/journal.pone.0044980 (PMC3459948; doi:10.1371/journal.pone.0044980)

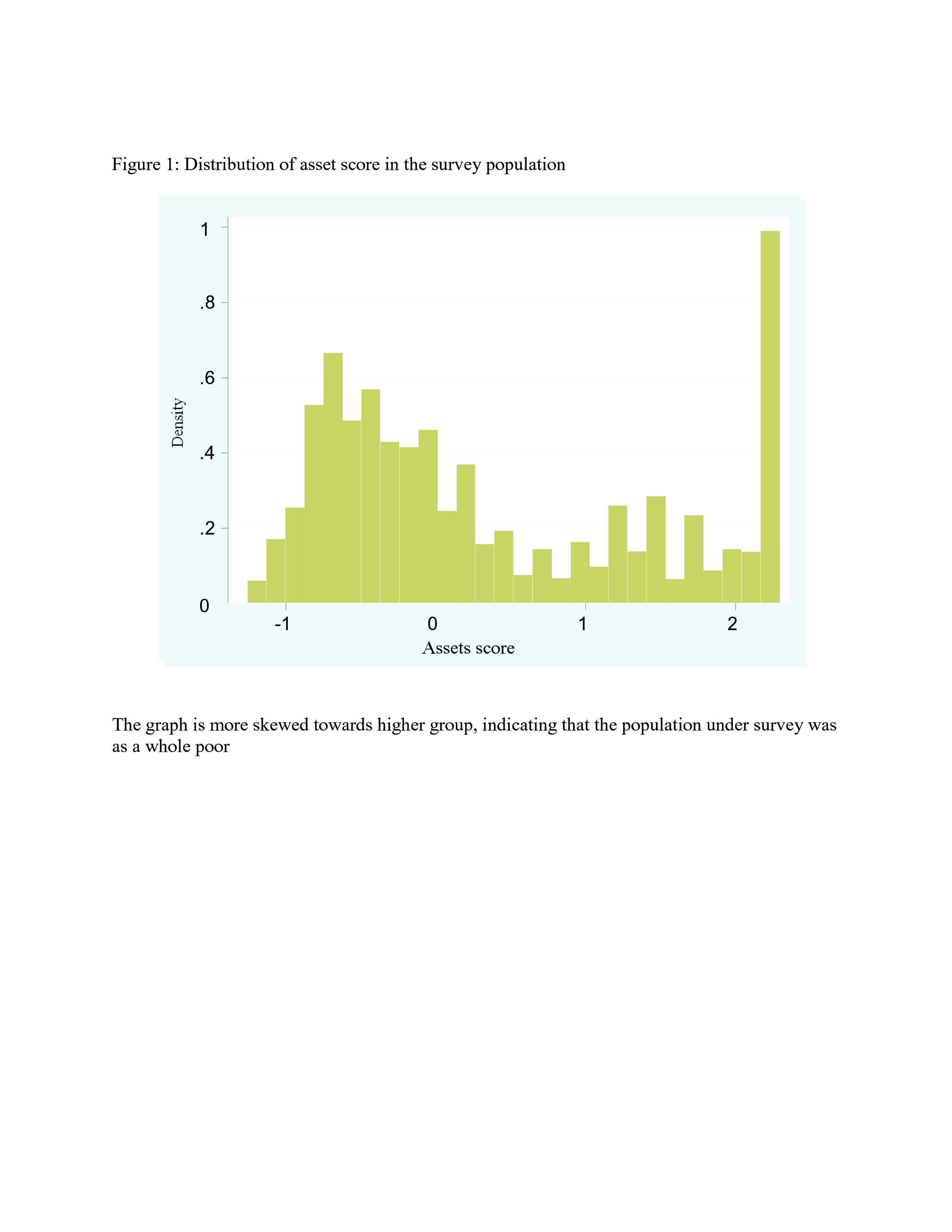

Supplement: Figure S1 — Distribution of asset score in the survey population. (TIF) [file pone.0044980.s001.tif]
